# Status of On‐Farm Artificial Insemination Services and Effectiveness of Oestrus Synchronization in Dairy Cattle Under Small‐Scale Dairy Farming System

**DOI:** 10.1002/vms3.71035

**Published:** 2026-06-16

**Authors:** Temesgen Milkias, Chencha Chebo, Alemayehu Worku

**Affiliations:** ^1^ Livestock and Fishery Development Office, Wolaita Zone South Ethiopia Ethiopia; ^2^ Department of Animal Sciences Arba Minch University, Arba Minch South Ethiopia Ethiopia

**Keywords:** dairy cattle, fertility, reproductive biotechnology, retrospective, smallholder farming

## Abstract

**Background:**

Hormonal synchronization of oestrous has been an available technology to improve the efficiency of field artificial insemination (AI) in the dairy sector.

**Objective:**

This study was aimed at investigating the breeding practices, status of AI and effectiveness of oestrus synchronization (ES) on small‐scale dairy farming in selected districts of the Wolaita Zone.

**Methods:**

Cross‐sectional survey and retrospective data on the oestrus synchronization were collected and analysed. Descriptive statistics, chi‐square tests, and indexed ranking procedures were applied to summarize the study data.

**Results:**

The predominant mating system practiced by dairy farmers was AI (55.6%), and 73.5% of farmers apply controlled mating. The highest response rate to PGF_2_α administration was obtained for cattle of Sodo town (89.5%), followed by Boloso Sore (78.8%). A 30%–45.2% pregnancy rate was obtained across the study districts. Crossbred cattle have shown significantly (*p* > 0.001) higher response (87.1%) and pregnancy rates (47.9%) than the local animals. Likewise, 9.6%–18.5% of the birth rate was obtained from this study. Farmers' overall perceptions and desire to continue using oestrus synchronization were improving as a result of their application experiences over time. Moreover, shortage of AI inputs and AI technicians, heat detection skills, and inconsistency of the AI service during festivals, holidays, and weekends were reported as the major challenges prevailing in the study districts.

**Conclusions:**

The study provided insights on improving input supply, accurate heat detection and intensified cattle management are necessities to enhance field AI services and increase pregnancy rates in synchronized animals.

## Graphical abstract

Cross‐sectional survey and retrospective data on the oestrus synchronization were collected and analysed. The predominant mating system practiced by dairy farmers was AI (55.6%), and 73.5% of farmers apply controlled mating. The highest response rate and pregnancy rate were 89.5% and 45.2%, respectively, whereas a birth rate of 9.6%–18.5% was obtained. Farmers' overall perceptions and desire to continue using oestrus synchronization were improving over time. The study provided insights on improving input supply; accurate heat detection and intensified cattle management are necessities to enhance AI services and increase pregnancy rates in synchronized animals.

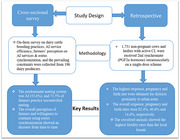

## 1 Introduction

Ethiopia has the untapped potential of cattle in Africa, having more than 70 million heads of cattle (CSA 2022). Artificial insemination (AI), an applied reproductive biotechnology, has been widely practiced for several decades in the country but has come with little success (Aynalem 2006; Dagmawit et al. 2022; Masho et al. 2024). Over the years, studies have reported that the absence of collaboration and regular communication between stakeholders, lack of breeding policy and herd recording system, inadequate resources, inputs and facilities and absence of incentives and rewards to motivate AI technicians have been reported as the major bottlenecks (Gizaw et al. 2016; Belete and Mulugeta 2021; Masho et al. 2024). Consequently, the reproductive performance of dairy animals has been declining with increasing numbers of days open and decreasing conception rates from year to year (Dagmawit et al. 2022; Masho et al. 2024).

On the other hand, to improve the efficiency of AI, hormonal synchronization of oestrous has been available and applied for more than three decades and used as a tool to make AI more practical (Sharew et al. 2023). Furthermore, hormonal oestrus synchronization (ES) is used for increasing the probability of oestrus detection, matching calving with feed availability and market demand for dairy products, and increasing pregnancy rates of dairy cattle (Azage et al. 2012; Patterson et al. 2016). It is also believed that ES programs improve reproduction efficiency by reducing the length of breeding and calving seasons, decreasing culling rates due to nonpregnant females, shortening calving intervals, and increasing calf weaning weights (Gupta et al. 2008; Graves 2009).

Oestrus synchronization in dairy cattle in Ethiopia was initiated in the late 1980s with the objectives of testing a simple hormonal ES regime and mass insemination under on‐farm conditions to improve access to improved dairy genetics by smallholder farmers and to kick‐start market‐oriented smallholder dairy development in selected sites (Azage et al. 2012). The initial objective was to improve access to improved dairy genetics by smallholder farmers and to enhance market‐oriented smallholder dairy development in Ethiopia (Azage et al. 2016). However, the performance of the scaled‐up project was inconsistent in the application of the technology and the results achieved (Gizaw et al. 2016). Consequently, in many regions of Ethiopia, farmers express low satisfaction with the service, although evaluation of the technology by farmers is confounded with several factors (Kebebew and Bekele 2018; Dagmawit et al. 2022; Sharew et al. 2023).

The previous studies conducted in the Wolaita Zone by Haben et al. (2020) and Hailemichael and Eshetu (2019) assessed AI services and the effectiveness of oestrus synchronization in a few dairy farms. However, these studies did not address the long‐term intervention of AI as well as oestrus synchronization in the study area. Furthermore, there are scant research findings on the evaluation of oestrus synchronization based on the retrospective data generated from the ES campaign program and farmers' perceptions. The study hypothesizes the long‐term practice of AI and oestrus synchronization might have a positive impact on enhancing dairy cattle breeding interventions. Therefore, the goal of this research was to assess the delivery status of AI and the effectiveness of ES in the study areas.

## 2 Material and Methods

### 2.1 Description of the Study Areas

The study was conducted in selected districts of the Wolaita Zone, Southern Ethiopia. The zone is composed of sixteen districts and six city administrations. The geographical location of the zone is at 37°10'0''E longitude with 60°20'0''N latitude. The zone has an annual temperature that varies between 24°C and 30°C and receives an average rainfall of 1350 mm per year. The Wolaita Zone is bordered on the south by Gamo, on the west by the Omo River, which separates Wolaita Zone from Dawro, on the northwest by Kembata Tembaro, on the north by Hadiya, on the northeast by the Oromia Region, on the east by the Bilate River, which separates it from the Sidama Region and on the southeast by Lake Abaya, which separates it from the Oromia Region.

### 2.2 Study Design and Duration

Cross‐sectional survey and retrospective study design were applied. The study was conducted from October 2022 to September 2023.

#### 2.2.1 Animals Selection and Management

Nonpregnant cows and heifers of indigenous and crossbreed animals were used in the study. A pregnancy test through rectal palpation was performed before hormone delivery to prevent abortion. Animals selected for hormonal synchronization were screened based on body condition (2 to 4 on a five‐scale dairy cattle body condition scoring basis), apparent health, breeding soundness, optimum body size, and absence of a fertility failure history. The selected animals were managed as per the farmer's routine husbandry system; that is, none of the animals were offered special feeding, housing, or healthcare management conditions. Due to better feed availability during April to May, oesrus synchronization campaigns were annually arranged in these months. The selected cows and heifers were ear‐tagged by the mobile team assigned for the annual campaign task.

#### 2.2.2 Protocols: Estrus Synchronization and Insemination

Through rectal palpation, animals with active CL were injected with 2 mL of PGF2α hormone intramuscularly on a single‐dose scheme. Prostaglandin hormone (synchromate, Bremer Pharma GmbH, Germany) was used during all years. Animals that come into standing heat within 3–5 days after hormone administration are inseminated according to the AM/PM principle within 6–12 h. Then, experienced AITs were recruited and assigned to the AI service. Frozen semen of Holstein Friesian, Jersey and Boran bulls, which was dispensed from the National Artificial Insemination Center, Kality, Addis Ababa were used for breeding animals. A total of 1731 animal records were obtained from the AI logbook from the years 2019 to 2022, of which 1048 were local, and 683 were crossbreed animals. Pregnancy diagnosis was achieved by rectal examination on Day 60 post‐insemination.

### 2.3 Data Collection

#### 2.3.1 Baseline Data

An on‐farm survey was conducted on breeding practices, AI service delivery systems, farmers’ perception of AI services and ES and the major constraints of the AI and ES. The researcher group held questionnaire‐based interviews and focused group discussions with cattle owners, model farmers, livestock production experts, AI technicians and animal health professionals in the respective districts. Primary data were generated through multi‐stage purposive sampling techniques. In the first stage, three rural districts and Sodo town were selected due to their pioneer utilization of AI and ES practices and for their dairy farming potential. In the second stage, six *kebeles* from Boloso Sore and Damot Gale districts and four *kebeles* from Sodo town and Humbo district were chosen using the same criteria. A total of 10 *kebeles* were selected, including two from highland, six from midland (major agroecology of the Wolaita Zone) and two from lowland agroecologies. Third, farmers with over 5 years of experience in both dairy production and adoption of AI and ES technologies received special consideration, with priority given to women dairy farmers. Cochran's (1977) proportional sample size determination formula was used to determine the number of households included in the survey (Equation 1).

(1) $$n=\frac{Z^{2}\left(p\right)\left(q\right)}{e^{2}}\;$$

where *n* refers to the desired sample size, *Z* is the standard normal deviation (1.96 for a 95% confidence level), *p* is 0.85 (estimated population variability proportion, 85%), *q* = 1‐p, that is, 0.15% or 15% and *e* = level of precision (0.05). Accordingly, 196 households—57 from Boloso Sore, 43 from Humbo, 52 from Damot Gale districts and 44 farmers from Sodo town—were considered for this study. Moreover, 98 farmers participated in the focused group discussion, which encompassed 8–10 farmers from each study *kebele*.

#### 2.3.2 Oestrus Synchronization Data Records

Only complete records from the AI logbook were screened and used. Data on the number of animals treated with hormones and responded, the number of services per conception, the pregnancy rate, and the number of calves born were used to evaluate the effectiveness of the oestrus synchronization practice. The effects of district, year, dam breed, body condition scores, and parity were considered as fixed effects. We have accessed and analysed synchronization data on 1731 animals with complete records.

### 2.4 Statistical Analysis

Descriptive statistics were employed to summarize the survey data. The chi‐square (*X* ^2^) test was used to check the relationship between the categorical variables. Constraints related to AI and oestrus synchronization were expressed by calculating an index according to the method described by Kosgey (2004) as follows: Index = the sum of (3 times first order + 2 times second order + 1 time the last order) given for an individual variable divided by the sum of (3 times first order + 2 times second order + 1 time the last order) for all variables. Moreover, the retrospective data on oestrus synchronization were entered into MS Excel and were analysed using SPSS software (SPSS, version 26). In Equations (2)–(5), the studied parameters were briefly defined. Response rate, pregnancy rate, and birth rate were analysed using frequency distribution and tested by the chi‐square test at a 5% significance level. Likewise, the number of services for conception was calculated according to Equation (3). The number of services per conception rate is calculated by dividing the total number of inseminated cows or heifers at a given time by the proportion of pregnancies confirmed by rectal examination at Day 60–90 post‐insemination. Moreover, the birth rate was calculated on the basis of initial animals administered the PGF_2_α hormone, as illustrated in Equation (5).

(2) $$\begin{array}{ccc} & & \mathrm{Response}\;\mathrm{rate}=\\ & & \;\frac{\mathrm{Number}\;\mathrm{of}\;\mathrm{animals}\;\mathrm{come}\;\mathrm{on}\;\mathrm{heat}\;\mathrm{following}\;\mathrm{hormone}\;\mathrm{injection}}{\mathrm{Total}\;\mathrm{number}\;\mathrm{of}\;\mathrm{animals}\;\mathrm{given}\;a\;\mathrm{hormone}\;}\;\times \;100 \end{array}$$

(3) $$\mathrm{NSPC}=\frac{\mathrm{Total}\;\mathrm{number}\;\mathrm{of}\;\mathrm{inseminations}\;\mathrm{until}\;\mathrm{animals}\;\mathrm{get}\;\mathrm{conceived}}{\mathrm{Total}\;\mathrm{number}\;\mathrm{of}\;\mathrm{cows}\;\mathrm{conceived}}\;$$

(4) $$\mathrm{Pregnancy}\;\mathrm{rate}=\frac{\mathrm{Number}\;\mathrm{of}\;\mathrm{animal}\;\mathrm{detected}\;\mathrm{positive}\;\mathrm{pregnancy}}{\mathrm{Total}\;\mathrm{number}\;\mathrm{of}\;\mathrm{animals}\;\mathrm{inseminated}}\;\times \;100$$

(5) $$\mathrm{Birth}\;\mathrm{rate}=\frac{\mathrm{Number}\;\mathrm{of}\;\mathrm{calves}\;\mathrm{produced}}{\mathrm{Total}\;\mathrm{number}\;\mathrm{of}\;\mathrm{animals}\;\mathrm{give}\;\mathrm{PGF}2\alpha \;\mathrm{hormone}\;}\;\times \;100$$

## 3 Results

### 3.1 Breeding Practices and AI Service Systems

Table 1 presents the situation analysis of current breeding practices and the status of AI service delivery in the study areas. The predominant mating system practiced by dairy farmers was AI alone (55.6%), followed by a combination of AI and bull mating (34.2%). There was a significant difference (*X* ^2^ = 18.13; *p* = 0.019) between districts regarding AI practices. Farmers in Sodo town (84.1%) and Damot Gale (78.8%) were found better in practising controlled mating than the other two districts. Most farmers (57.2%) have more than 20 years of experience practising AI as a dairy improvement strategy. However, 80.6% of farmers reported that they could not access the AI service regularly. Interruptions occurred during weekends and holidays (79.6%), and necessary facility limitations also affected efficient AI service access.

**TABLE 1:** Breeding practices and AI services status in study districts.

Variables	Boloso Sore *N* (%)	Humbo *N* (%)	Damot Gale *N* (%)	Sodo town *N* (%)	Overall *N* (%)	Chi‐square	*p*‐value
Commonly used mating system						31.85	0.008
Only bull mating	5 (8.8)	8 (18.6)	4 (7.7)	3 (6.8)	20 (10.2)		
Only AI	15 (26.3)	23 (53.5)	35 (67.3)	36 (81.8)	109 (55.6)		
Both	37 (64.9)	12 (27.9)	13 (25.0)	5 (11.4)	67 (34.2)		
Do you practice a controlled mating?						18.13	0.019
Yes	40 (70.2)	26 (60.5)	41 (78.8)	37 (84.1)	144 (73.5)		
No	17 (29.8)	17 (39.5)	11 (21.2)	7 (15.9)	52 (26.5)		
How long have you been using AI service?						14.52	0.021
5–10 years	3 (5.3)	15 (34.9)	5 (9.6)	2 (4.5)	25 (17.8)		
10–20 years	41 (71.9)	18 (41.9)	38 (73.1)	15 (34.1)	112 (57.2)		
Above 20 years	13 (22.8)	10 (23.3)	9 (17.3)	27 (61.4)	59 (30.0)		
Do you access AI in regular basis?						24.40	0.001
Yes	8 (14.0)	4 (9.3)	6 (11.5)	20 (45.5)	38 (19.4)		
No	49 (86.0)	39 (90.7)	46 (88.5)	24 (54.5)	158 (80.6)		
Where and how do you get AI service?						7.23	0.005
At AI centre	48 (84.2)	38 (88.4)	48 (92.3)	28 (63.6)	162 (82.7)		
Calling AITs to my farm‐gate	9 (15.8)	5 (11.6)	4 (7.7)	16 (36.4)	34 (17.3)		
AITs round at community on scheduled days	0 (0.0)	0 (0.0)	0 (0.0)	0 (0.0)	0 (0.0)		
Is AI service available during weekends and holidays?						26.95	0.006
No	5 (8.8)	3 (7.0)	5 (9.6)	27 (61.4)	40 (20.4)		
Yes	52 (91.2)	40 (93.0)	47 (90.4)	17 (38.6)	156 (79.6)		
What do you do if you lack AITs when your animals are in heat?						39.22	0.001
Take animals to bull service	26 (45.6)	10 (23.3)	29 (55.8)	34 (77.3)	99 (50.5)		
Wait for the next oestrous cycle	22 (38.6)	27 (62.8)	19 (36.5)	6 (13.6)	74 (37.8)		
Do nothing	9 (15.8)	6 (14.0)	4 (7.7)	4 (9.1)	23 (11.7)		
What do you do if your animals do not conceive with repeated AI services?						10.05	0.018
Cull the animals by selling	26 (45.6)	13 (30.2)	24 (46.2)	7 (15.9)	70 (35.7)		
Continue on giving AI service	15 (26.3)	9 (20.9)	15 (28.8)	29 (65.9)	68 (34.7)		
Use a bull mating	16 (28.1	21 (48.8)	13 (25.0)	8 (18.2)	58 (29.6)		
Do AITs check the conception status of your AI served animals?						9.05	0.027
Yes	5 (8.8)	4 (9.3)	8 (15.4)	32 (72.7)	49 (25.0)		
No	52 (91.2)	39 (90.7)	44 (84.0)	12 (27.7)	147 (75.0)		
Do you know the breeding history of your crossbred animals?						17.14	0.011
Yes	3 (5.3)	0 (0.0)	5 (9.6)	12 (27.3)	20 (10.2)		
No	54 (94.7)	43 (100)	47 (90.4)	32 (72.7)	176 (89.8)		

This study showed that a significantly higher proportion of dairy farmers access AI services by taking their animals to AI centres (82.7%) located at agricultural offices. In contrast, those who live near AI services, such as farmers in Sodo town (36.4%), call AITs to their farm‐gate when animals are in heat. During weekends and holidays, when AITs are inaccessible, the majority of farmers instead take animals to bull service (50.5%), followed by those who wait for the next heat cycle (37.8%). If animals do not conceive after repeated service, the owners either cull animals by selling (35.7%) or continue by giving AI service (34.7%). Notably, except in Sodo town, AITs rarely conduct pregnancy diagnosis and follow‐up of AI‐served animals during the gestation period. Furthermore, most farmers (89.8%) have limited knowledge of the breeding and pedigree history of their crossbred animals, such as blood type, success and failure records of previous inseminations and the overall AI impact; instead, they focus only on milk yield increments due to crossbreeding.

### 3.2 Farmers Participation and Status of Oestrus Synchronization Programme in the Study Areas

The majority of farmers (83.7%) are not actively involved in the main activities of the yearly oestrus synchronization campaign (Table 2). Most of the oestrus synchronization activities were carried out by AITs, zonal and district livestock production experts at a scheduled time, once a year. In addition, 65.3% of farmers have limited knowledge about how animals are selected for synchronization. About one‐fourth (24.5%) of farmers reported a failure of AIs on synchronized animals. Of these, 12.8% and 9.2% reported that AI service failures occurred once and twice, respectively.

**TABLE 2:** Farmer's perception and status of oestrus synchronization programme across the study districts.

Variables	Boloso Sore *N* (%)	Humbo *N* (%)	Damot Gale *N* (%)	Sodo town *N* (%)	Overall *N* (%)	Chi‐square	*p*‐value
Are you actively participating in the programme?						18.43	0.022
Yes	6 (10.5)	3 (7.0)	5 (9.6)	18 (40.9)	32 (16.3)		
No	51 (89.5)	40 (93.0)	47 (90.4)	26 (59.1)	164 (83.7)		
Do you know how animals are selected for estrus synchronization?						13.11	0.004
Yes	15 (26.3)	11 (25.6)	13 (25.0)	29 (65.9)	68 (34.7)		
No	42 (73.7)	32 (74.4)	39 (75.0)	15 (34.1)	128 (65.3)		
Do you face any failure of AI services on synchronized animals?						6.45	0.127
Yes	12 (21.1)	10 (23.3)	14 (26.9)	12 (27.3)	48 (24.5)		
No	45 (78.9)	33 (76.7)	38 (73.1)	32 (72.7)	148 (75.5)		
How many times have you observed insemination failure after oestrus synchronization?						3.73	0.682
None	45 (78.9)	33 (76.7)	38 (73.1)	32 (72.5)	148 (75.5)		
Once	7 (12.3)	5 (11.6)	7 (13.5)	5 (11.3)	25 (12.8)		
Twice	5 (8.8)	4 (9.3)	5 (9.6)	4 (9.1)	18 (9.2)		
Three times	0 (0.0)	1 (2.3)	3 (6.9)	1 (2.3)	5 (2.5)		
Do you access better AI service as compared to only conventional AI?						15.64	0.013
Yes	40 (70.2)	28 (65.1)	35 (67.3)	34 (77.3)	137 (69.9)		
No difference	17 (29.8)	15 (34.9)	17 (32.7)	10 (22.7)	59 (30.1)		
What do you do if you lack AITs when your animals are in heat?						22.87	0.001
Take animals to bull service	38 (66.7)	31 (72.1)	39 (75.0)	12 (27.3)	120 (61.2)		
Wait for the next oestrus cycle	14 (24.6)	8 (18.6)	11 (21.1)	29 (65.9)	62 (31.6)		
Do nothing	5 (8.8)	4 (9.3)	2 (3.8)	3 (6.8)	14 (7.1)		
What is your overall interest in oestrus synchronization programme?						12.19	0.008
High	22 (38.6)	15 (34.9)	15 (28.8)	22 (50.0)	74 (37.8)		
Medium	27 (47.4)	23 (53.5)	29 (55.8)	16 (36.4)	95 (48.5)		
Low	8 (14.0)	5 (11.6)	8 (15.4)	5 (11.4)	26 (13.3)		
From your experience, which programme do you interested to use in future?						5.25	0.066
Conventional AI alone	15 (26.3)	11 (25.6)	12 (23.1)	9 (20.5)	47 (24.0)		
AI with estrus synchronization	35 (61.4)	23 (53.5)	34 (65.4)	30 (68.2)	122 (62.2)		
Bull service	7 (12.3)	9 (20.9)	6 (11.5)	5 (11.4)	27 (13.8)		

Abbreviations: AI, artificial insemination; AITs, artificial insemination technicians.

The majority (69.9%) of farmers believe that they access a better AI service during a synchronization campaign than the routine AI service. In situations similar to the conventional AI process, when AITs are unavailable, farmers are forced to take oestrous‐synchronized animals to bull service and then wait for the next oestrous cycle. Regarding perception, about 48.5% of dairy farmers have a medium level of perception towards utilization of the oestrus synchronization programme, while 37.8% have rated it highly. Due to better perception and experiences gained through time, the majority (62.2%) of farmers reported that they prefer to continue using AI services jointly with oestrus synchronization. Furthermore, a significantly (*p* < 0.05) higher proportion of dairy farmers in Sodo town were aware of and participated in the synchronization programme, and showed better performance.

### 3.3 Constraints Related With AI and Oestrus Synchronization

Table 3 presents the most commonly prevailing constraints in the districts associated with AI and oestrus synchronization. Accordingly, shortages of sustainable AI services and field facilities; heat detection and timing of insemination; interruption of services during weekends and holidays; lack of incentives and refreshment training for AITs; and poor husbandry management practices were reported constraints associated with AI services in the study areas, as a ranked order. During the group discussion, farmers and experts noted that the lack of monitoring the progress of AI impact and post‐semen quality evaluation would impair the efficiency of AI services in the study areas. Moreover, lack of double injection of PGF2α, following further reproductive status of synchronized animals, wrong animal selection, weak participation of farmers in all steps of the programme, and poor husbandry management practices were ranked in order of importance.

**TABLE 3:** Ranking of constraints related with AI and estrus synchronization in the study districts.

Reported constraints	1st	2nd	3rd	Index	Rank
Constraints related with AI services					
Heat detection and timing of insemination problem	39	36	31	0.16	2
Interruption of AI service during weekends and holidays	40	33	36	0.16	2
Shortage of continues semen and field AI facilities	49	31	27	0.17	1
Poor dairy cattle husbandry management	40	32	28	0.15	3
Lack of incentives and refreshment training for AITs	45	23	36	0.15	3
Lack of post semen quality evaluation	26	20	21	0.09	5
Lack of monitoring, evaluation and progress recording	33	14	25	0.11	4
Constraints related to oestrus synchronization					
Lack of double shot option	47	42	33	0.23	1
Lack of follow‐up after hormone administration	48	35	32	0.22	2
Wrong animal screening/selection	36	34	35	0.19	3
Weak involvement of farmers	38	37	27	0.19	3
Poor husbandry management	36	34	26	0.18	4

### 3.4 Effectiveness of Oestrus Synchronization

#### 3.4.1 Response Rate to PGF2α Hormone Treatment

As presented in Table 4, except for year (*X* ^2^ = 4.36; *p* = 0.225), all other factors had a significant (*p* < 0.001) effect on the response rate of cows and heifers. The overall response rate obtained from the current study was 82.4%. Regarding the district effect, the obtained response rate ranged from 77.7% to 89.5%, with the highest being recorded from Sodo town (89.5%) and the lowest being from Humbo district. Crossbreed animals have shown a higher response rate (87.12%) than the indigenous cattle types. Furthermore, cows of parities two to four had the highest conception rate, while heifers and first parity cows had the lowest. Slight progress was observed across the 4‐year interval of the synchronization programme; however, it did not show a statistically significant change. The body condition also did not show a significant (*X* ^2^ = 8.94; *p* = 0.061) difference.

**TABLE 4:** Response rate of animals treated with PGF2α hormone in the study districts.

Effects	PGF2α treated (*N*)	Responded(*N*)	Response rate (%)	*X* ^2^ test	*p*‐value
Districts				21.5	0.001
Boloso Sore	538	434	80.7^b^		
Damot Gale	544	430	79.0^b^		
Humbo	260	202	77.7^c^		
Sodo town	389	348	89.5^a^		
Breed				23.2	0.001
Local	1048	819	78.2^b^		
Crossbreed	683	595	87.1^a^		
Parity				18.7	0.001
Heifers	410	287	70.2^c^		
1st parity	675	542	80.3^b^		
2nd and 3rd parity	535	487	91.0^a^		
4th and above	111	98	88.3^a^		
BCS				8.94	0.061
2	1397	1128	80.7		
3	276	236	85.5		
4	58	50	86.2		
Year				4.36	0.225
2019	441	350	79.4		
2020	463	380	82.1		
2021	381	307	80.6		
2022	446	377	84.5		
Overall	1731	1414	82.4		

*Note: N* refers number of animals received hormone and responded.

#### 3.4.2 Number of Inseminations and Pregnancy Rate

The success of oestrus synchronization mainly depends on regularly monitoring and inseminating animals at the optimal in‐heat time, as well as the number of animals that conceive during the first service. As indicated in Table 5, all factors except year had a significant (*p* < 0.05) effect on pregnancy rate. The overall average number of services per conception was 1.48. Notably, animals in the Humbo district, indigenous breeds, and heifers required more repeated inseminations compared to their counterparts. In contrast, a higher pregnancy rate was observed among crossbreed animals, cows in their 2nd to 3rd parity and cows with a body condition score of 2 and 3, based on the five‐point dairy cattle body condition scoring method. Furthermore, across the study districts, the pregnancy rate among hormone‐treated animals ranged from 40.9% to 52.1%, with the highest rate observed in Sodo town and the lowest in the Humbo district.

**TABLE 5:** Inseminations and pregnancy rate of synchronized animals.

Effects	Inseminated (*N*)	NSPC (mean)	Pregnancy (+ve, *N*)	Pregnancy rate (%)	*X* ^2^	*p*‐value
Districts					35.7	0.001
Boloso Sore	281	1.35	123	43.8^b^		
Damot Gale	247	1.42	118	47.8^b^		
Humbo	127	1.79	52	40.9^c^		
Sodo town	263	1.21	137	52.1^a^		
Breed					42.86	0.001
Local	461	1.69	185	40.1^b^		
Crossbreed	457	1.22	245	53.6^a^		
Year					5.32	0.503
2019	208	1.46	91	43.8		
2020	245	1.61	109	44.5		
2021	201	1.52	97	48.3		
2022	264	1.40	133	50.4		
Parity					51.9	0.001
Heifers	216	1.76	86	39.8^c^		
1st parity	320	1.55	148	46.3^b^		
2nd and 3rd parity	310	1.36	162	52.3^a^		
4th parity and above	72	1.44	34	47.2^b^		
BCS					38.2	0.001
2	711	1.48	318	44.7^b^		
3	169	1.33	97	57.4^a^		
4	38	1.46	15	39.5^c^		
Overall	918	1.50	430	46.6		

*Note*: NSPC refers number of services per conception. +ve denotes positive response.

#### 3.4.3 Birth Rate on Basis of PGF2α‐Treated Animals

As presented in Table 6, significant differences (*p* < 0.05) were observed between districts, parity, breed and body condition score for the number of hormone‐administered animals, calves born and birth rate per hormone‐treated animal. Accordingly, the overall birth rate value obtained was 14.4% (i.e., 245 calves were produced out of 1731 animals treated). Similar to other response variables, Sodo town had the highest birth rate, followed by the Boloso Sore district. The 2nd and 3rd parity cows, those with a BCS of 2, and crossbreed animals had a higher birth rate than their counterparts. Likewise, animals synchronized in 2022 had better birth rates than in other years. Furthermore, most animals were categorized as having an ‘unknown’ status due to missing data after hormonal treatment, while a few were recorded as aborted and sold.

**TABLE 6:** Birth rate (%) on the basis of hormone‐treated animals.

Variables	Hormone‐treated animals (*N*)	Calf born (*N*)	Aborted (*N*)	Sold (*N*)	Unknown (*N*)	Birth rate per hormone treated (%)
District						
Boloso Sore	538^a^	76^b^	5	13	47	14.1^b^
Damot Gale	544^a^	72^b^	7	12	46	13.2^b^
Humbo	260^c^	25^c^	2	6	27	9.6^c^
Sodo town	389^b^	72^a^	3	9	65	18.5^a^
Parity						
Heifers	410^b^	52^c^	5	10	34	12.7^b^
1st parity	675^a^	102^b^	4	13	46	15.1^b^
2nd and 3rd parity	535^a^	77^a^	5	10	85	14.4^a^
4th and above	111^c^	14^c^	3	7	20	12.6^a^
Year						
2019	441	45	6	7	53	10.2
2020	463	59	4	11	67	12.7
2021	381	64	3	7	54	16.8
2022	446	77	4	15	64	17.3
Breed						
Local	1048^a^	110^b^	10	28	75	10.5^b^
Crossbreed	683^b^	135^a^	7	12	110	19.8^a^
BCS						
2	1397^a^	90^a^	7	21	140	12.7^b^
3	276^b^	40^b^	4	16	37	21.7^a^
4	58^c^	5^c^	6	3	8	12.1^b^
Overall	1731	245	17	40	185	14.4

Source of variations	*p*‐values		
District	0.001	0.012	0.008
Parity	0.003	0.001	0.001
Year	0.154	0.067	0.138
Breed	0.001	0.001	0.001
Body condition score	0.001	0.001	0.001

*Note: N* denotes number of animals. *p*‐values refer significance values tested at 5%.

## 4 Discussion

### 4.1 Breeding Practices and AI Service Efficiency

Recently, dairy farming has become the main source of family income and a government priority area in Ethiopia; consequently, farmers are shifting from the uncontrolled natural breeding system to the most profitable venture, AI and oestrus synchronization. Moreover, better access to AI services, coupled with the intensification of dairy farming, the gradual diminishing of grazing areas, and high demand for dairy products, encouraged dairy farmers to exercise controlled AI mating. This was preferably observed in Sodo town and the Boloso Sore and Damot Gale districts in the current study. Similarly, several studies reported that AI and AI with synchronization were used more frequently in Ethiopia; as a result, farmers have a high tendency to breed their animals through mating with improved genotypes in several areas of Ethiopia (Destalem 2015; Gizaw et al. 2016; Kebebew and Bekele 2018; Dagmawit et al. 2022).

On the contrary, due to a lack of organized AI facilities and field equipment and incentives for AITs, there were high interruptions of AI services, particularly during weekends and holidays. This long‐term application had not been documented to have a significant impact. This has been previously noted by Bainesagn (2015), who reported that in West Shoa, only 4.5% of the respondents reported receiving regular AI service; the remaining either received irregular service (90.4%) or did not practice AI (5.1%). The majority of people in Andracha (56.7%) and Masha (72.5%) did not receive AI services on a regular basis (Masho et al. 2024). Similarly, Ahmed et al. (2017) reported that only 34.2% of smallholder dairy producers in Debre‐Tabor had access to AI services on a regular and uninterrupted basis, whereas 65.8% did not.

In addition, as observed in this study, Roelofs et al. (2006), Azage et al. (2016), Kebebew and Bekele (2018), and Belete and Mulugeta (2021) reported several problems associated with the existing AI system at the national level, such as technical limitations, lack of transport facilities, poor quality of semen, poor heat detection, lack of incentive, and unavailability of the service off‐working hours (weekends, holidays, etc.). The problem has been more aggravated by the lack of private sectors rendering this service, since AI service is primarily provided by the government, which accounts for 99.1% of the insemination service in Ethiopia (Bainesagn 2015). Moreover, regular AI service supply was hampered by poor infrastructure, road access, and limited transportation services (Masho et al. 2024).

Consequently, these reports indicate that the majority of farmers were forced to take animals to bull services, passing without breeding animals, waiting for the next oestorus cycle, and doing nothing when the AI service was inaccessible. Accordingly, in the Oromia region of Ethiopia, 53.7% of farmers either look for natural mating or skip the mating period and wait for the next oestrous cycle (41.1%) (Bainesagn 2015). About 58.8% of dairy farmers in Hadiya zone, Ethiopia, take animals to natural mating; 13.8% were reported passing the estrus time without breeding and do nothing (36.4%) (Masho et al. 2024). Similarly, most of the farmers in Oromia (60.5%) use AI service at AI stations, and 68.6% of farmers in the Tigray study use on‐call service (Destalem 2015). Regarding service access, the majority of farmers access AI services by taking their animals to AI centers. However, farmers located in and around towns call artificial inseminators to their farm gates, and the rest wait for the next oestrous cycle.

Culling through selling is a customized practice by most farmers when animals fail to conceive after several services. However, if those animals have a good breeding history, farmers keep trying AI up to three to four times. Moreover, we noticed that there was no pregnancy diagnosis carried out by AI technicians after insemination, and farmers do not know clear breeding histories, such as blood level, genetic group, or duration of key reproductive permanence indicator traits of their crossbred animals. This might be due to AITs sometimes breeding cows with Jersey or Holstein bull semen with different blood levels, and in the next mating, the previous mating history is not commonly referred to. Farmers also have no experience in keeping records for common performance traits.

### 4.2 Farmers' Participation and Status of Oestrus Synchronization

In the study districts, at the beginning of the synchronization programme, the majority of farmers were rejecting it due to low heat detection and pregnancy rates, but gradually, farmers are adopting it better. Currently, farmers are accepting oestrus synchronization technology as a good opportunity to improve the reproductive efficiency of their animals. Consequently, the overall farmers’ satisfaction was rated as low to medium, with the majority of farmers expressing better satisfaction. Moreover, dairy farmers residing near service access areas reported high or complete satisfaction, indicating that they had better perceptions than rural farmers. In addition, the study revealed that there was a higher desire to continue using oestrus synchronization. Similarly, studies conducted in Oromia, SNNP, and Tigray indicated that farmers’ satisfaction with hormonal estrus synchronization technology is gradually increasing due to improvements in the pregnancy rates and calf crop delivery achievements following hormone treatments (Gizaw et al. 2016).

### 4.3 Constraints Related With AI and Oestrus Synchronization

#### 4.3.1 Constraints Related With AI Services

As observed in the current study, the traditional AI service has been facing a continued shortage of semen and liquid nitrogen supply and field AI facilities under on‐farm conditions (Debir 2015; Gizaw et al. 2016; Dagmawit et al. 2022). Likewise, poor heat detection and timely inseminations, lack of post‐semen quality examinations, and interruptions of services during weekends and holidays were remarkable challenges observed in the study. Poor routine management practices, such as a shortage of quality and sufficient nutrition, reproductive care, and welfare services for high‐grade cows, were also common problems affecting the fertility of animals and the success of the programme. More importantly, few field AI technicians were giving services to wide geographical areas encompassing many beneficiary farmers without encouraging incentives and refreshing in‐service training. Consequently, the efficiency of AI service in Ethiopia has been found to be low (Desalegn et al. 2009). This might be due to technical limitations, lack of transport facilities, poor quality of semen, poor heat detection, lack of incentive, and unavailability of the service off‐working hours, which are constraining the AI system in Ethiopia (Azage et al. 2012; Dagmawit et al. 2022). Moreover, short duration, low intensity, and poor expression of oestrus signs in Ethiopian Zebu cattle could be the cause of most oestrous detection failures observed in the smallholders' condition (Mukassa‐Mugerwa et al. 1989; Bekele et al. 1991; Gizaw et al. 2016).

#### 4.3.2 Constraints Related to Oestrus Synchronization

*Lack of double‐dose synchronization*: Studies reporting that a higher oestrus response and conception rates were obtained among cows and heifers that received double injection (Gizaw et al. 2016). Moreover, Tadesse (2015) observed that the odds of oestrus response in cows and heifers that received a double injection were 2.6 times more likely to respond to the hormone when compared to animals that received a single injection. Similarly, Chanyalew et al. (2017) showed that higher pregnancy rates were obtained in the double injection of PGF_2_α treatment (63.1%) than in cows treated with a single‐shot protocol (55.8%). Likewise, Sharew et al. (2023) reported a higher conception rate for a double shot (100%) than a single shot (90.8%) for dairy cows in central Ethiopia. However, Gizaw et al. (2016) recommended that the single‐dose protocol with accurate heat detection could be a feasible option for smallholder farming conditions in Ethiopia.

*Lack of continuous follow‐up*: Similarly, several studies reported that the lack of follow‐up after hormone administration was the most important constraint of the oestrus synchronization scheme under small‐scale farmers. This was due to the assigned professionals, including the AITs, being engaged during the short synchronization and insemination of those coming into heat within 2–5 days. After that, animals are not regularly followed for the whole gestation process, such as pregnancy and fetal health diagnosis, embryonic losses, calving preparations and birth rate, and there is no platform for reporting those events to the concerned offices.

*Weak farmers' participation and wrong animal screening*: Successful calf production in a hormone‐synchronized oestrus and AI breeding system is determined by the identification of cycling cows/heifers with a functional corpus luteum, accurate heat detection (standing heat), a timely and correct insemination procedure, and avoiding factors causing embryo and fetal mortality (Gizaw et al. 2016; Dagmawit et al. 2022). However, in the current study, due to the rapid action of the campaign schedule, we noticed less involvement of farmers and a high chance of wrong animal screening, which had a highly significant impact on the outcome, as indicated in the low birth rate. In addition, the wrong perception of experts that reporting a high number of animals synchronized and responding to the hormone during the campaign is a success indicator has misled policymakers to overlook the true problems prevailing on the ground.

*Poor husbandry management*: Poor management of synchronized animals has been reported to have a negative impact on the desired success of the programme. Even though the synchronization scheme was adjusted to the moisture available in the seasons, assuming sufficient feed availability, it was not well supported by providing quality feed, controlling breeding systems, and ensuring healthy and comfortable housing conditions. Similarly, Wubneh (2020), Belete and Mulugeta (2021), and Sharew et al. (2023) reported that poor estrus detection and management of dairy cattle hinder the oestrus synchronization effort.

### 4.4 Effectiveness of Oestrus Synchronization

#### 4.4.1 Response Rate and Number of Services per Conceptions

Due to proximity to services, the oestrus response obtained in Sodo town and Boloso Sore districts is relatively higher than in other districts. The overall response rate (82.4%) obtained from this study was higher than the 57.7% in the Awassa‐Dale milkshed and 61.7% in the Adigrat‐Mekelle milkshed reported by Azage et al. (2016). Likewise, higher response rates were reported in Ethiopian smallholder dairy farms by Masho et al. (2024), who reported 78.5%; Wubneh et al. (2020), who reported 91.2% in South Omo; and Tegegn and Zelalem (2017), who reported 89% in Southwest Ethiopia. Azage et al. (2012) reported 97.7% in the Hawassa‐Dale milkshade area and 100% in the Adigrat‐Mekelle milkshade area. Kebede et al. (2013) reported an oestrus rate of 89.3% in the Bahir Dar milkshed; 72.3% and 92.17% oestrus rates were reported in the West Shoa zone by Bainesagn (2015) and Girmay et al. (2015) in the Wukro Kilte Awulaelo district in Northern Ethiopia, respectively. Moreover, using the same protocol with the current study, an 84.2% oestrus rate was reported in the eastern zone of the Tigray region, Ethiopia (Tadesse 2015).

Oestrus response of cows/heifers to hormonal treatment varied with breeds. On average, a higher percentage of exotic crossbred cows/heifers (86.7%) than local cows/heifers (78.4%) responded to treatment (Gizaw et al. 2016). This could be due to the fact that estrus duration in exotic breeds is longer, so the response to hormone treatment is higher. Similarly, Plasse et al. (1970) reported that the duration of sexual receptivity in *Bos taurus* females varied from 4 to 48 h (mean value between 13.60 and 19.30 h), while in *Bos indicus* cows, the mean duration of oestrous was short (6.70 h), which also varied from 2 to 22 h. The number of services per conception (1.5 times) obtained was much lower than 2.3 times, as reported by Masho et al. (2024). The variations observed might be due to several factors, such as the breed of animals, reproductive management, heat detection, and the skill of inseminators.

#### 4.4.2 Pregnancy and Calving Rates

The ultimate outcomes expected in animal breeding are the number of calves born and conception rate, and successful pregnancy until 60 days post‐AI was also considered as evaluation criterion (Gizaw et al. 2016). However, including the current study, most of the oestrus synchronization programmes result in high response and low conception rates. Similarly, Wubneh et al. (2020) reported an 11.02% pregnancy rate in South Omo; Masho et al. (2024) reported 43.3% in the Hadiya zone. A lower average conception rate (27.1% at the national level and 33.3% at the Southern regional level) was reported by Azage et al. (2016). Girmay et al. (2015) obtained a 32.17% pregnancy rate in the Awulaelo District of Northern Ethiopia; Tegegn and Zelalem (2017) observed a 24.7% pregnancy rate in the Mizan Aman area of Southwest Ethiopia; and Desalgn (2008) reported the average national conception rate of 27% in Ethiopia. On the other hand, the conception rates found in Bangladesh (46.2%), reported by Shamsuddin et al. (2001); 54.3% in Dakar, Senegal, by Abonou (2007); 51.5% by Hossain et al. (2016) in Bangladesh; and 57.3% by Paul et al. (2011) in the Sirajgonj district were higher than the current report.

The observed disparities in pregnancy rate might be due to inappropriate timing of insemination, errors associated with detection of estrus, body condition of the animal, nutrition and management, early embryonic death, insemination techniques, skill of artificial inseminators, proper semen thawing procedure, placement of semen in the uterus, quality and handling techniques of semen, reproductive disorders, long calving interval, and age of the cow (Gizaw et al. 2016; Tegegn and Zelalem 2017; Chanyalew et al. 2017).

In line with our observation, Chanyalew et al. (2017) reported a declining trend in the pregnancy rate after the third parity might be related to lactation stress and the older cows reducing the chance of fertility and tending to gain weight. The higher pregnancy rate obtained for exotic animals was in contrast with higher conception rates for local cows/heifers, higher (77.4%) than for the exotic crossbred cows (68.8%) in the Oromia region (Bainesagn 2015). However, our report was in line with exotic crosses having higher percentages (68.4%) than local animals (53.3%) in Southern Ethiopia (Debir 2015). Likewise, Sharew et al. (2023) reported higher response and conception rates for crossbred than for indigenous cattle. This might be due to their genetic variations and reproductive physiological soundness.

Animals with body condition scores of 2 and 3 had a higher rate of pregnancy (44.7% and 57.4%, respectively) compared to cows/heifers with a body condition score of 4 (39.5%). Similarly, an optimal body condition for maximum conception rate appears to be around a body condition score of 4.5 in the Amhara study (Samuel 2015), 4 in Tigray (Tadesse 2015), and 2 in the Oromia study (Bainesagn 2015). Animals must be in a better physical condition or on a nutritional gain plan. This includes suitable quantities of dry matter, as well as protein, minerals, and vitamins in particular. Poor reproductive performance is generally connected with a poor or high body condition score. Moreover, Dagmawit et al. (2022) reported that mucus flow from external genitalia, noticeable uterine tone, good body condition, and lactating cows had a significant effect on conception rate.

The large group of animals categorized as unknown might be anestrous cows or prepubertal heifers since they will not respond to an injection of PGF_2_α since no CL exists (Gizaw et al. 2016). Similarly, Dagmawit et al. (2022) reported that anoestrus (30.5%) and repeat breeders (38.9%) were among the causes of the low conception rate during the synchronization programme. Further challenges to the oestrus synchronization and AI programme are embryo loss (which was found to be high in the current study); incidence of missed AI opportunity due to failure to detect heat and wrong insemination of non‐oestrous cows, and pregnancy diagnosis through rectal palpation, which could be intrusive and could not be done earlier than 60 days post‐AI (Gizaw et al. 2016). A higher embryo loss of 15%, 25%, 40%, and 30% was determined in the Tigray, Amhara, Oromia, and SNNP experiments, respectively (Tadesse 2015; Samuel 2015; Bainesagn 2015; Debir 2015).

Similar to our findings, a high rate of embryo death, ranging from 15% to 40%, was reported by Gizaw et al. (2016). This might be due to when prostaglandin is injected into animals with silent estrus and unidentified pregnancy, it causes embryo loss (Santos et al. 2004), so before synchronization, the animal should be checked for pregnancy by sensitive diagnostic equipment such as ultrasound, progesterone profiling using different enzymes, ELISA, and a detection kit for pregnancy‐related glycoprotein. Furthermore, Smith et al. (2011) reported that factors affecting embryonic loss are numerous and include genetic abnormalities, fescue toxicosis, plant toxins, excess protein, heat stress, reproductive diseases, and the effect of the sire, and handling or transportation stresses.

## 5 Conclusions

The study identified the existence of a well‐growing trend and the shifting of a traditional, uncontrolled breeding system to more advanced techniques like AI and hormonal synchronization in the study districts. On the other hand, several challenges related to AI and oestrus synchronization utilization were reported at the farmer's level. In contrast, farmers have shown better adoption of oestrus synchronization technology over time and have a positive desire to improve the reproductive efficiencies of dairy animals. Thus, the study provided good insight to strengthen the field AI service and improve pregnancy and birth rates in synchronized dairy animals through the supply of field inputs, proper heat detection, and intensified overall management systems.

## Author Contributions

**Temesgen Milkias**: conceptualization, investigation, methodology, data curation, validation, formal analysis, writing – original draft. **Chencha Chebo**: conceptualization, investigation, methodology, data curation, software, formal analysis, validation, supervision, writing – review and editing. **Alemayehu Worku**: data curation, validation, supervision, writing – review and editing.

## Funding

The authors have nothing to report.

## Ethics Statement

We confirm that the ethical policies of the journal, as noted on the journal's author guidelines page, have been adhered to, and that appropriate ethical review committee approval was obtained from the Livestock and Fisheries Research Center (approval number AMU/LFRC/0240/22, dated 08 October 2022). All procedures were conducted in accordance with Directive 2010/63/EU of the European Parliament and of the Council of 22 September 2010 on the protection of animals used for scientific purposes, to ensure that the methods used did not cause the animals avoidable pain, suffering, distress, or lasting harm.

## Conflicts of Interest

The authors declare no conflicts of interest.

## Data Availability

The datasets used for this study will be available from the corresponding author upon reasonable request.
